# Aberrant microstructural integrity of white matter in mild and severe orthostatic hypotension: A NODDI study

**DOI:** 10.1111/cns.14586

**Published:** 2024-02-08

**Authors:** Yingzhe Cheng, Lin Lin, Shaofan Jiang, Peilin Huang, Jiejun Zhang, Jiawei Xin, Haibin Xu, Yanping Wang, Xiaodong Pan

**Affiliations:** ^1^ Department of Neurology, Center for Cognitive Neurology Fujian Medical University Union Hospital Fuzhou City China; ^2^ Fujian Institute of Geriatrics Fujian Medical University Union Hospital Fuzhou City China; ^3^ Institute of Clinical Neurology Fujian Medical University Fuzhou City China; ^4^ Fujian Key Laboratory of Molecular Neurology Fujian Medical University Fuzhou City China; ^5^ Department of Radiology Fujian Medical University Union Hospital Fuzhou City China; ^6^ Fujian Key Laboratory of Intelligent Imaging and Precision Radiotherapy for Tumors Fujian Medical University Fuzhou City China; ^7^ Center for Geriatrics Hainan General Hospital Hainan China; ^8^ Fujian Medical University Fuzhou City China; ^9^ Department of Endocrinology Fujian Medical University Union Hospital Fuzhou City China

**Keywords:** cognition, MOH, NODDI, plasma biomarker, SOH, WM integrity

## Abstract

**Objective:**

Scarce evidence is available to elucidate the association between the abnormal microstructure of white matter (WM) and cognitive performance in patients with orthostatic hypotension (OH). This study investigated the microstructural integrity of WM in patients with mild OH (MOH) and severe OH (SOH) and evaluated the association of abnormal WM microstructure with the broad cognitive domains and cognition‐related plasma biomarkers.

**Methods:**

Our study included 72 non‐OH (NOH), 17 MOH, and 11 SOH participants. Across the groups, the WM integrity was analyzed by neurite orientation dispersion and density imaging (NODDI), and differences in WM microstructure were evaluated by nonparametric tests and post hoc models. The correlations between WM microstructure and broad cognitive domains and cognition‐related plasma biomarkers were assessed by Spearman's correlation analysis.

**Results:**

The abnormal WM microstructure was localized to the WM fiber bundles in MOH patients but distributed widely in SOH cohorts (*p* < 0.05). Further analysis showed that the neurite density index of the left cingulate gyrus was negatively associated with amyloid β‐40, glial fibrillary acidic protein, neurofilament light chain, phospho‐tau181 (*p* < 0.05) but positively with global cognitive function (MOCA, MMSE, AER‐III), memory, attention, language, language fluency, visuospatial function and amyloid β‐40 / amyloid β‐42 (*p* < 0.05). Additionally, other abnormal WM microstructures of OH were associated with broad cognitive domains and cognition‐related plasma biomarkers to varying degrees.

**Conclusion:**

The findings evidence that abnormal WM microstructures may present themselves as early as in the MOH phase and that these structural abnormalities are associated with cognitive functions and cognition‐related plasma biomarkers.

## Graphical abstract

The abnormal WM microstructure was localized to the WM fiber bundles in MOH patients but distributed widely in SOH cohorts. Moreover, these abnormal WM microstructures with broad cognitive domains and cognition‐related plasma biomarkers. The findings may provide novel insights into the impact of abnormal WM microstructure on the cognition of OH patients, suggesting the necessity of early intervention in clinical practice.

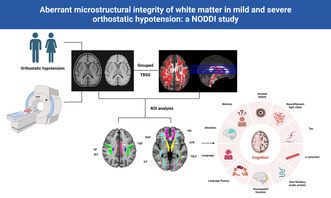

## 1 INTRODUCTION

Orthostatic hypotension (OH), an important manifestation of autonomic nervous dysfunction,^1^ usually occurs when the cardiovascular regulatory mechanism fails to compensate for the reduced venous return due to the upright posture of the body, reflecting sympathetic innervation or reflex regulation disorder. Recently, a host of clinical studies have demonstrated a close implication of OH in cardiovascular diseases and cognition‐related diseases, and its close correlation with poor prognosis in the elderly.^2^, ^3^, ^4^ However, scarce literature is available to investigate the white matter (WM) microstructure in OH patients and its connection to the broad cognitive domain and cognition‐related plasma biomarkers.

OH is defined as a decrease in systolic blood pressure (SBP) of at least 20 mmHg or a decrease in diastolic blood pressure (DBP) of at least 10 mmHg within 3 min during a postural switch from decubitus to orthostatic or an upright tilt test.^1^ Most available studies of the correlation between OH and cognitive function have referred to the above typical definition in their OH diagnosis. Other studies have focused on the meticulous differences and classified patients with multisystem atrophy into mild OH (MOH) (an SBP decrease of 20–30 mmHg or DBP decrease of 10–15 mmHg) and severe OH (SOH) (an SBP decrease of 30 mmHg or DBP decrease of over 15 mmHg).^5^ Given the scarcity of relevant literature, the current study adopted this classification criterion and grouped the participants into MOH and SOH patients to elucidate the potential effects of OH on cognition.

In current neuroimaging, diffusion tensor imaging (DTI) is a classical diffusion magnetic resonance imaging (dMRI). However, its efficacy has been compromised due to two basic limitations.^6^, ^7^ First, the DTI diffusion index from a single diffusion‐weighted *B*‐value shell and a simple three‐dimensional Gaussian model is an average measurement of water diffusion from multiple compartments (e.g., extracellular and intracellular spaces), which may have different diffusivities, shapes, and orientations. Second, the DTI does not adequately describe water diffusion in the cross, kiss, and fan‐shaped fiber regions. Recent studies have documented that the incidence of cross fibers in the cerebral WM is about 90%, supporting that the pathological specificity of DTI indicators in complex WM regions of the brain is significantly lower than expected.^7^, ^8^, ^9^ Alternatively, neurite orientation dispersion and density imaging (NODDI), a multicompartment biophysical model of dMRI, can potently differentiate the measurements of the cerebrospinal fluid, extracellular fluid, and intracellular fluid.^10^ Therefore, compared with DTI, NODDI is more sensitive to the density of neurites and the degree of fiber dispersion, and can delineate the changes in tissue microstructure, providing a window into the anatomical basis of brain functions.^11^ A recent study reveals a high consistency of the neurite density index (NDI) of NODDI with histological measurements of neurite compartment density in isolated mouse brains.^12^ Therefore, compared with those of DTI, NODDI indexes may offer a higher specificity in explaining diffusion‐weighted microstructural features in clinical imaging of human brains.

Therefore, the current study employed NODDI to analyze the WM integrity in the OH population (aged 50 years and over) by recruiting 72 non‐OH (NOH), 17 MOH, and 11 SOH adults, and to investigate its correlation with cognition and cognition‐related plasma biomarkers. The findings may provide novel insights into the impact of abnormal WM microstructure on the cognition of OH patients, suggesting the necessity of early intervention in clinical practice.

## 2 METHODS

### 2.1 Ethical approval statement

This study was approved by the Ethics Committee of Fujian Medical University Union Hospital, Fuzhou City, Fujian Province (Ethics No.: 2021KJCX040). This study was conducted in accordance with the principles of the Declaration of Helsinki.

### 2.2 Study subjects

**FIGURE 1:**
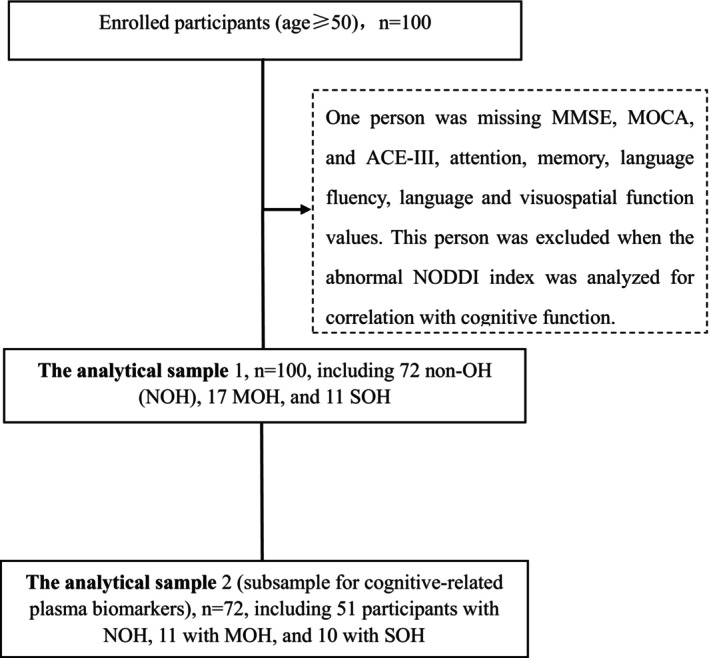
Flowchart of the participant recruitment. ACE‐III, Addenbrooke's Cognitive Examination version III; MMSE, mini‐mental state examination; MOCA, Montreal Cognitive Assessment; MOH, mild orthostatic hypotension; NODDI, Neurite orientation dispersion and density imaging; SOH, severe orthostatic hypotension.

The study included all participants over 50 years of age who attended the cognitive psychology clinic of Fujian Medical University Union Hospital between May 2022 and February 2023. Participants underwent diffusion‐weighted imaging (DWI) on the day they completed the neuropsychological assessment. All participants provided written informed consent at the time of study registration and DWI data collection. For participants with severe cognitive impairment, the informed consent was obtained from their guardians. The flowchart of participant recruitment is depicted in Figure 1.

### 2.3 Data collection and assessment

Data were collected through face‐to‐face interviews, clinical examinations, and neuropsychological questionnaires by neurologists with more than 5 years of experience in neurology. The data covered a range of potential factors, including age, sex, education, BP, medication history, history of stroke or other neurological diseases, and cognitive performance. Hypertension was defined as SBP ≥ 140 mmHg, DBP ≥ 90 mmHg, or the use of antihypertensive medication. Stroke is defined based on records or typical neurological symptoms or MRI findings.

### 2.4 Assessment of the cognitive function

Global cognition was assessed using mini‐mental state examination (MMSE),^13^ Montreal Cognitive Assessment (MOCA),^14^ and Addenbrooke's Cognitive Examination version III (ACE‐III) scales.^15^ The latter was used to assess cognitive function in five subdomains, including attention, memory, language, verbal fluency, and visuospatial function.^15^

### 2.5 Determination of plasma biomarkers

Blood samples were collected into EDTA‐coated vacuum containers, which were then centrifuged to obtain plasma. The centrifuged blood samples were stored at −80 ° C and thawed immediately before composition quantification. The levels of plasma amyloid β‐42 (Aβ42), amyloid β‐40 (Aβ40), glial fibrillary acidic protein (GFAP), neurofilament light chain (NFL), phospho‐tau181 (*p*‐tau) and α‐synuclein were measured using the Human Neurology 3‐Plex A Assay (N3PA) kit on an automatic single‐molecule‐array (SIMOA) instrument (Quanterix Corp, MA, USA) according to the manufacturer's protocol.

### 2.6 Assessment of the MOH and SOH

All participants were instructed to have a light diet on the day of assessment. All BP tests were performed at noon to decrease the impact of circadian rhythms on hemodynamics and were evaluated in a quiet room by the same cardiovascular physician. All BP tests were performed with a fully automatic electronic sphygmomanometer (Omron HBP‐1300; Omron Healthcare, Dalian, China). The BP was recorded after a 5‐min rest and a second measurement was performed immediately after the first assessment. The average of the two measurements was taken as the supine BP value. The participants were then asked to stand up as soon as possible and their orthostatic BP was measured immediately at 0, 1, 2, and 3 min. MOH was defined as an SBP drop of 20–30 mmHg or a DBP drop of 10–15 mmHg within 3 min of standing. SOH was designated as an SBP decrease of more than 30 mmHg or a DBP decrease of more than 15 mmHg within 3 min of standing.^5^ NOH was defined as a decrease in SBP of less than 20 mmHg and a decrease in DBP of less than 10 mmHg within 3 min of the posture change from supine to orthostatic position.^5^ The ΔSBP represents the SBP after standing minus the SBP when lying on the back. Similarly, ΔDBP represents the DBP after standing minus the DBP when lying on the back. Negative values indicate lower blood pressure after standing.

### 2.7 Acquisition of magnetic resonance imaging (MRI) data

MRI data acquisition was performed using a 3 T magnetic resonance scanner with a 64‐channel receive‐only head coil (MAGNETOM Prisma, Siemens Healthcare, Erlangen, Germany). The foam was placed around the head of each participant before the test to reduce head movement. All participants were asked to refrain from moving their heads during the MRI scan. The B value was set at 0, 1000, 2000, and 3000 s/mm^2^ (scanned in 95 directions); 72 slices with a thickness of 2 mm were used. The other scan parameters were as follows: repetition time (TR), 5800 ms; time to echo (TE), 91 ms; field‐of‐view (FOV), 215 × 215 mm^2^; generalized autocalibrating partially parallel acquisition, 2; slice acceleration factor, 2; number of averages, 1; voxel size, 2 × 2 × 2 mm^3^, without gap; and acquisition time, 9 min 44 s. High‐resolution T1‐weighted images were acquired using a Magnetization Prepared‐RApid Gradient Echo (Mprage) sequence with the following parameters: TR, 2300 ms; TE, 2.32 ms; FOV, 240× 240 mm^2^; number of averages, 1; voxel size, 0.9 × 0.9 × 0.9 mm^3^,192 slices; and acquisition time, 4 min 44 s.

### 2.8 MRI preprocessing and analysis

The image quality was checked for image parameters, the number of gradient directions, the signal‐to‐noise ratio, head motion, artifacts, etc., and then the original data meeting the requirements were subjected to the following preprocessing: (1) Data format conversion, using dcm2nii software to convert DICOM format into nii.gz format; (2) The FDT (FMRIB's Diffusion Toolbox, FDT) eddy correction in FSL (FMRIB Software Library, FSL) for head motion eddy current and gradient direction correction; (3) The acquisition of the b0 image of brain mask; and (4) the acquisition of the neurite density index (NDI),^16^ the orientation dispersion index (ODI) and the isotropic volume fraction (FISO)^17^ scalar index via the dtifit function in FSL.

Tract‐Based Spatial Statistics (TBSS) analysis was conducted with the FSL software. The main steps were as follows: (1) the original individual FA map was registered to the MNI standard space (Montreal Neurological Institute space); (2) an average FA skeleton was constructed with an FA value >0. 2; (3) the individual FA diagram was projected onto the average FA skeleton to obtain the individual skeleton. Similarly, the NDI, ODI, and FISO skeleton diagrams were constructed based on the FA skeleton; and (4) the randomized tool in FSL was used to conduct double independent sample *t*‐test and replacement test between the two groups (replacement times 5000 times), and the differences in the NDI, ODI and FISO values of the WM between the two groups were detected. Multiple comparison correction was performed by the threshold‐free cluster enhancement (TFCE) method to extract the indicators of different brain areas.

The aberrant WM skeletal regions with statistical differences in TBSS analysis were labeled with reference to JHU White‐Matter Tractography Atlas.^18^ The number of impaired voxels in different JHU regions was calculated and the percentage of damaged voxels was obtained. To avoid circular analysis, the top 3 JHU regions with the largest percentage of impaired voxel numbers were considered areas of interest (ROI). Finally, the mean values of NDI, ODI, and FISO were calculated for the 3 JHU ROI skeletons.

### 2.9 Statistical analyses

SPSS 26 software was used for the statistical analysis. Statistical significance was defined as a two‐sided *p* < 0.05. Data were tested for normal distribution by the Kolmogorov–Smirnov test. Continuous variables conforming to normal distribution were expressed as mean ± standard deviation and analyzed by *t*‐test. The non‐normal distribution variables were expressed as the median (quartile) and compared by a non‐parametric test; categorical variables were expressed as numbers (percentages) and compared by a chi‐square test. The extracted NODDI indexes were tested nonparametrically among the three groups and further analyzed by the post‐hoc test corrected by Bonferroni in case of statistical significance. Finally, their correlations with cognition and plasma biomarkers were respectively assessed by Spearman's correlation analysis. One person was excluded from the correlation analysis due to a lack of data, including ACE‐III, MMSE, MOCA, memory, attention, language fluency, language, and visuospatial function. A *p*‐value of <0.05 was considered statistically significant.

## 3 RESULTS

### 3.1 Clinical and demographic characteristics of participants

Sample cohort 1 included 72 NOH, 17 MOH, and 11 SOH participants, who reported no significant differences in gender, age, education, stroke, ACE‐III, MMSE, MOCA, memory, attention, language fluency, language, and visuospatial function (*p* > 0.05), but significant differences in hypertension, supine SBP, supine DBP, ΔSBP, and ΔDBP among the three groups (*p* < 0.01) (Table 1). Sample cohort 2 included 51 NOH, 11 MOH, and 10 SOH participants, who showed no significant differences in gender, age, education, hypertension, stroke, ACE‐III, MMSE, MOCA, memory, attention, language fluency, language, visuospatial function, Aβ42, Aβ40, *p*‐tau, GFAF, NFL, Aβ42/Aβ40, and α‐synuclein (*p* > 0.05), but statistically significant differences in hypertension, supine SBP, supine DBP, ΔSBP, and ΔDBP among the three groups (*p* < 0.05) (Table 2).

**TABLE 1:** Characteristics of the study participants (Sample cohort 1).

Characteristics^a^	NOH (*n* = 72)	MOH (*n* = 17)	SOH (*n* = 11)	*p* overall
Age	64.23 ± 7.67	64.35 ± 7.29	69.64 ± 10.89	0.176
Female (*n*, %)	46 (73.00)	11 (17.50)	16 (9.50)	0.826
Education(years)	10.66 (5.20)	11.71 (5.10)	10.00 (4.77)	0.817
Hypertension (*n*, %)	7 (9.70)	6 (35.30)	7 (63.60)	<0.001***
Stroke (*n*, %)	13 (81.30)	3 (18.80)	0 (0.00)	0.308
MMSE	28.00 (5.00)	28.00 (7.00)	28.00 (8.00)	0.876
MOCA	27.00 (10.00)	28.00 (8.00)	26.00 (9.00)	0.574
ACE‐III	86.00 (26.00)	87.00 (16.00)	85.00 (26.00)	0.459
Attention	18.00 (2.00)	18.00 (3.00)	18.00 (5.00)	0.883
Memory	20.00 (11.00)	21.00 (8.00)	19.00 (16.00)	0.371
Language fluency	9.00 (5.00)	10.00 (5.00)	8.00 (6.00)	0.643
Language	24.00 (6.00)	24.00 (3.00)	24.00 (9.00)	0.407
Visuospatial function	15.00 (4.00)	15.00 (2.00)	15.00 (4.00)	0.472
Supine hemodynamics				
Supine SBP, mmHg	117.00 (23.00)	127.00 (25.00)	143.00 (13.00)	<0.001***
Supine DBP, mmHg	74.00 (13.00)	82.00 (9.00)	82.00 (13.00)	0.002**
Supine heart rate, bpm	66.00 (13.00)	68.00 (14.00)	67.00 (17.00)	0.857
Change on standing				
ΔSBP, mmHg	7.00 (19.00)	−22.00 (3.00)	−32.00 (11.00)	<0.001***
ΔDBP, mmHg	7.00 (8.00)	−4.00 (15.00)	−15.00 (24.00)	<0.001***
Δheart rate, bpm	9.00 (9.00)	8.00 (12.00)	8.00 (5.00)	0.798

Abbreviations: ACE‐III, Addenbrooke's Cognitive Examination version III; DBP, diastolic blood pressure; MMSE, mini‐mental state examination; MOCA, Montreal Cognitive Assessment; MOH, mild orthostatic hypotension; SBP, systolic blood pressure; SOH, severe orthostatic hypotension; ΔDBP, change in orthostatic diastolic pressure; Δheart rate, change in orthostatic heart rate; ΔSBP, change in orthostatic systolic blood pressure.

*Note*: Data were presented as number (percentage), median (quartile).

^a^ One person was missing MMSE, MOCA, and ACE‐III, attention, memory, language fluency, language and visuospatial function values. ***p < 0.001, and **p < 0.01.

**TABLE 2:** Characteristics of the study participants (Sample cohort 2).

Characteristics	NOH (*n* = 51)	MOH (*n* = 11)	SOH (*n* = 10)	*p* overall
Age	64.0 (11.00)	63.0 (9.00)	72.5 (17.00)	0.317
Female (*n*, %)	32 (62.74)	7 (63.64)	5 (50.00)	0.738
Education(years)	11.0 (5.00)	11.0 (7.00)	9.5 (8.00)	0.755
Hypertension (*n*, %)	7 (13.2)	4 (36.36)	6 (60.00)	0.003**
Stroke (*n*, %)	9 (90.00)	1 (10.00)	0 (0.00)	0.297
MMSE	29.00 (4.00)	28.00 (7.00)	29.00 (7.00)	0.834
MOCA	27.00 (7.00)	26.00 (8.00)	26.5 (8.00)	0.562
ACE‐III	89.00 (27.00)	87.00 (19.00)	85.00 (26.00)	0.602
Attention	18.00 (2.00)	18.00 (1.00)	18.00 (4.00)	0.989
Memory	21.00 (10.00)	21.00 (9.00)	19.00 (11.00)	0.641
Language fluency	9.00 (6.00)	10.00 (4.00)	8.50 (5.00)	0.671
Language	25.00 (4.00)	24.00 (4.00)	24.00 (9.00)	0.182
Visuospatial function	15.00 (3.0)	15.00 (4.00)	15.0 (3.00)	0.918
Supine hemodynamics				
Supine SBP, mmHg	117.00 (23.00)	129.00 (27.00)	141.50 (14.00)	<0.001***
Supine DBP, mmHg	74.00 (14.00)	81.00 (11.00)	81.50 (15.00)	0.024*
Supine heart rate, bpm	67.00 (14.00)	68.00 (14.00)	68.00 (20.00)	0.749
Change on standing				
ΔSBP, mmHg	7.00 (21.00)	−22.00 (2.00)	−31.50 (11.00)	<0.001***
ΔDBP, mmHg	6.00 (9.00)	−3.00 (14.00)	−13.50 (25.00)	0.001**
Δheart rate, bpm	10.00 (9.00)	10.00 (13.00)	7.50 (6.00)	0.571
Aβ42	6.51 (1.95	6.10 (1.64)	7.26 (3.14)	0.401
Aβ40	117.92 (41.10)	114.05 (19.64)	117.72 (51.35)	0.776
*p*‐tau	2.33 (2.00)	1.99 (0.81)	2.18 (1.868)	0.911
GFAP	82.15 (95.94)	83.84 (25.55)	117.56 (109.22)	0.759
NFL	16.99 (18.60)	16.33 (9.00)	27.01 (32.20)	0.087
Aβ42/ Aβ40	0.06 (0.01)	0.05 (0.02)	0.05 (0.02)	0.607
α‐synuclein	11,103.42 (15,495.85)	5596.80 (17,990.01)	7090.97 (15,911.66)	0.298

*Note*: Data were presented as number (percentage), median (quartile).

Abbreviations: ACE‐III, Addenbrooke's Cognitive Examination version III; Aβ40, Amyloidβ‐40; Aβ42, Amyloidβ‐42; GFAP, glial fibrillary acidic protein; MMSE, mini‐mental state examination; MOCA, Montreal Cognitive Assessment; MOH, mild orthostatic hypotension; NFL, Neurofilament light chain; *p*‐tau, phospho‐tau181; SBP, systolic blood pressure; DBP, diastolic blood pressure; SOH, severe orthostatic hypotension; ΔDBP, change in orthostatic diastolic pressure; Δheart rate, change in orthostatic heart rate. ***p < 0.001; **p < 0.01; *p < 0.05.

### 3.2 The results of TBSS analysis (Sample cohort 1)

**FIGURE 2:**
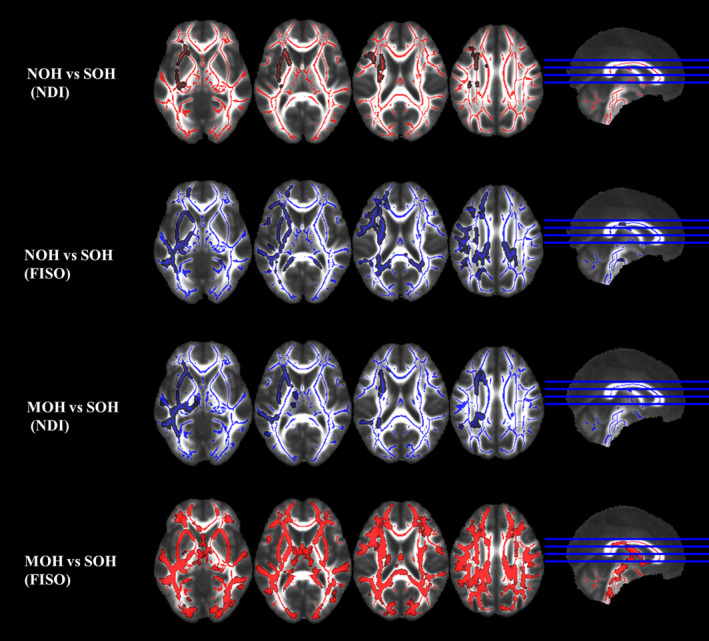
The results of diffusion metrics by tract‐based spatial statistics (TBSS). FISO, isotropic volume fraction; MOH, mild orthostatic hypotension; NDI, neurite density index; SOH, severe orthostatic hypotension.

TBSS statistical analysis showed significant differences in NDI and FISO in several cerebral regions between NOH and SOH groups and between MOH and SOH groups, including the left inferior fronto‐occipital fasciculus (IFO), left anterior thalamic radiation (ATR), left inferior longitudinal fasciculus (ILF), left superior longitudinal fasciculus (SLF), left superior longitudinal fasciculus–temporal part (TSLF), left uncinate fasciculus (UF), left cingulum (cingulate gyrus, CgC) and forceps minor (FM), which were hereinafter referred to as comprehensive abnormal fiber bundles (*p* < 0.05), whereas, no statistically significant difference in ODI was evident between them (*p* > 0.05). In addition, no statistically significant differences in NDI, ODI, and FISO were found between the NOH and MOH groups (*p* > 0.05) (Figure 2; Tables 3 and 4).

**TABLE 3:** Anatomical regions of tract‐based spatial statistics results (NOH vs. SOH).

NOH vs. SOH	Cluster Index	Voxels	*p*	Anatomical regions (Top 3)	MAX *X* (mm)	MAX *Y* (mm)	MAX *Z* (mm)
NDI	4	4350	0.035	Inferior fronto‐occipital fasciculus L:6.08713 Anterior thalamic radiation L:6.00897 Inferior longitudinal fasciculus L:4.38069	−20	16	−13
	3	22	0.05	Superior longitudinal fasciculus L:58.6818 Superior longitudinal fasciculus (temporal part) L:35.1818 Inferior fronto‐occipital fasciculus L:0.272727	−35	−23	26
	2	5	0.05	Superior longitudinal fasciculus L:9.6	−46	−41	36
	1	4	0.05	Superior longitudinal fasciculus L:14.75	−43	−39	34
FISO	6	16,738	<0.001	Inferior fronto‐occipital fasciculus L:3.26759 Superior longitudinal fasciculus L:3.08287 Inferior longitudinal fasciculus L:2.91809	−25	29	9
	5	41	0.049	Superior longitudinal fasciculus L:78.6341 Superior longitudinal fasciculus (temporal part) L:36.8537	−37	−35	31
	4	24	0.048	Forceps minor:32.2917 Inferior fronto‐occipital fasciculus L:15.125 Uncinate fasciculus L:12.3333	−13	48	−14
	3	9	0.049	Uncinate fasciculus L:0.666667	−17	6	−15
	2	8	0.05	Cingulum (cingulate gyrus) L:10.75	−15	−57	29
	1	3	0.05	Superior longitudinal fasciculus L:72 Superior longitudinal fasciculus (temporal part) L:50	−35	−28	27

Abbreviation: FISO, isotropic volume fraction; MOH, mild orthostatic hypotension; NDI, neurite density index; SOH, severe orthostatic hypotension.

**TABLE 4:** Anatomical regions of tract‐based spatial statistics results (MOH vs. SOH).

MOH vs. SOH	Cluster index	Voxels	*p*	Anatomical regions (Top 3)	MAX *X* (mm)	MAX *Y* (mm)	MAX *Z* (mm)
NDI	1	7825	0.02	Inferior fronto‐occipital fasciculus L:7.33125 Inferior longitudinal fasciculus L:5.38633 Anterior thalamic radiation L:4.90006	−24	12	−13
FISO	1	49,740	0.002	Forceps minor:1.90816 Inferior fronto‐occipital fasciculus L:1.80583 Superior longitudinal fasciculus L:1.75332	26	−52	18

Abbreviation: FISO, isotropic volume fraction; MOH, mild orthostatic hypotension; NDI, neurite density index; SOH, severe orthostatic hypotension.

### 3.3 ROI analysis results based on the TBSS analysis (Sample cohort 1)

**FIGURE 3:**
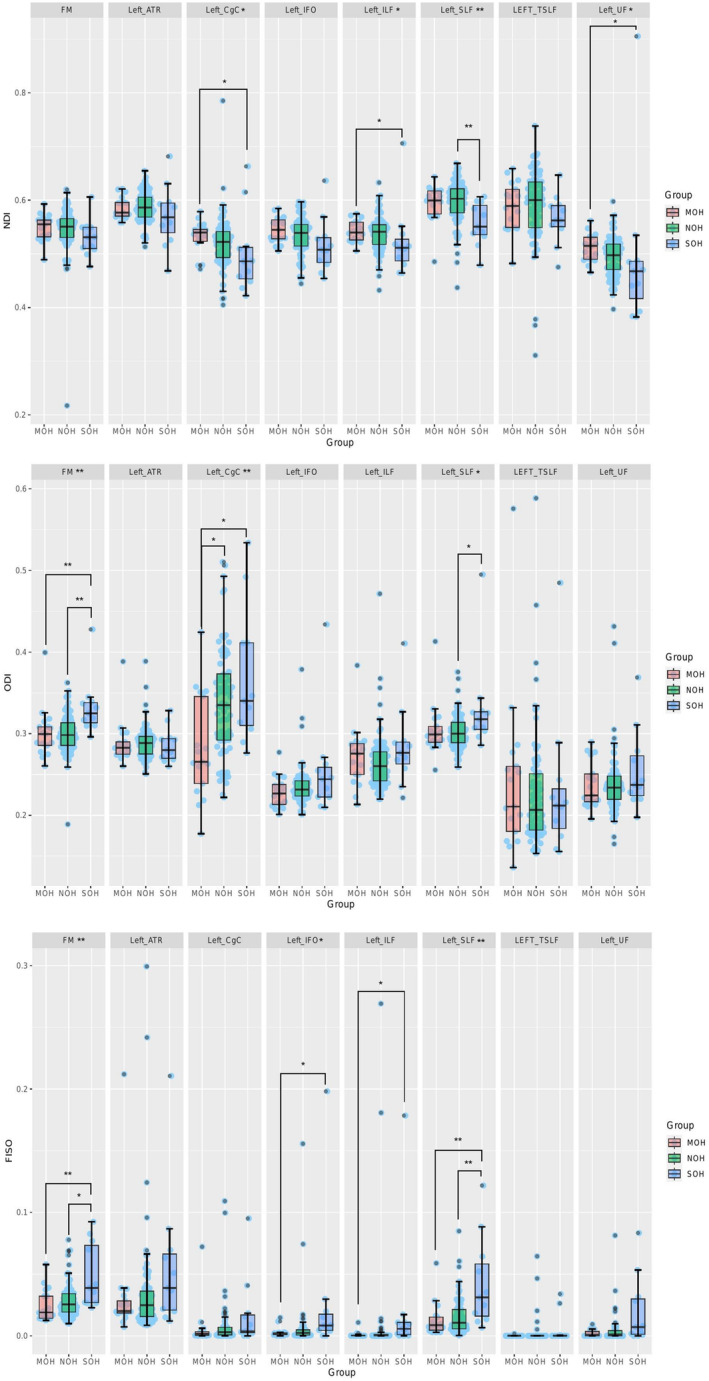
The results of important fiber bundles in the region of interest (ROI) by cluster‐based spatial statistics (TBSS). Different boxplot represents different diffusion metrics. MOH, mild orthostatic hypotension; NOH non‐orthostatic hypotension; SOH, severe orthostatic hypotension.

Compared with the NOH group, ODIs of the left SLF and FM in the SOH group increased to different degrees; ODIs of the left CgC in the MOH group decreased to varying degrees. Compared with the MOH group, ODIs of the left CgC and FM in the SOH group increased to different degrees; NDIs of the left ILF, left UF and left CgC in the SOH group were reduced to different degrees; however, the FISOs of the above fiber bundles and the left IFO in the SOH group increased to varying degrees. The NDIs and FISOs of the left SLF in the SOH group were significantly lower than those in the NOH group. In addition, no significant differences in other fiber bundle indicators were observed between the groups (*p* < 0.05) (Figure 3).

### 3.4 Association of aberrant NODDI indicators with cognition (Sample cohort 1)

**FIGURE 4:**
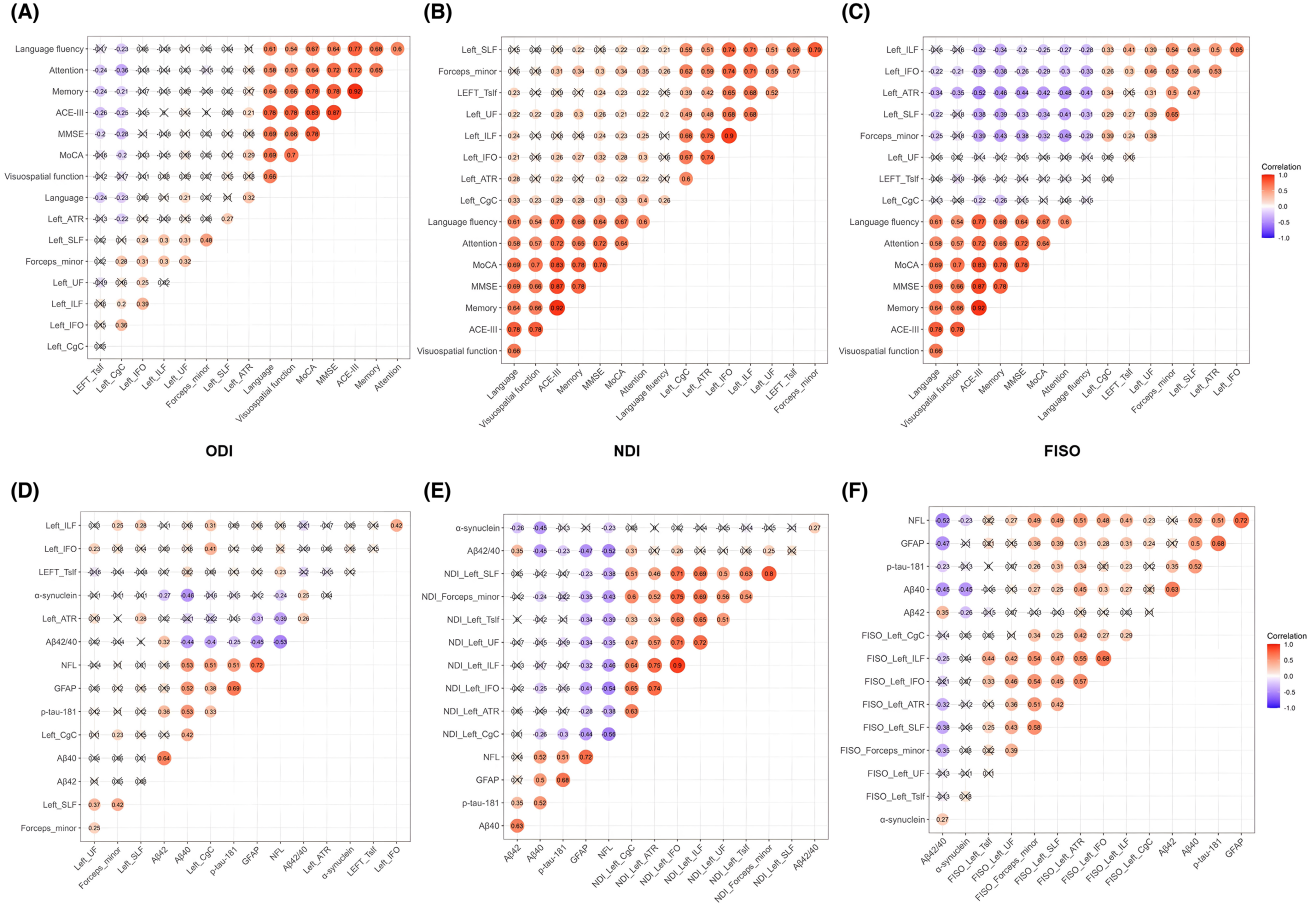
The correlation results between diffusion metrics with cognitive function and cognition‐related biomarkers. (A) Correlation between the ODI of abnormal fiber bundles and cognitive function. (B) Correlation between the NDI of abnormal fiber bundles and cognitive function. (C) Correlation between the FISO of abnormal fiber bundles and cognitive function. (D) Correlation between the ODI of abnormal fiber bundles and cognition‐related plasma biomarkers. (E) Correlation between the NDI of abnormal fiber bundles and cognition‐related plasma biomarkers. (F) Correlation between the FISO of abnormal fiber bundles and cognition‐related plasma biomarkers. FISO, isotropic volume fraction; NDI, neurite density index; ODI, orientation dispersion index.

The ODI of left ATR was positively associated with ACE‐III, MOCA, and language scores, while that of left UF was positively associated with language scores. Nevertheless, the ODI of left CgC was negatively associated with ACE‐III, MMSE, MOCA, memory, attention, language fluency, and language scores, and that of left TSLF was negatively associated with ACE‐III, MMSE, MOCA, memory, attention, and language scores (*p* < 0.05) (Figure 4A).

The NDI of left UF, and left CgC were positively associated with ACE‐III, MMSE, MOCA, memory, attention, language fluency, language, and visuospatial function scores, and those of left ILF and left TSLF were positively with the MMSE, MOCA, attention, and language scores. The NDI of left IFO was positively associated with ACE‐III, MMSE, MOCA, memory, attention, and language scores. The NDI of FM was positively associated with ACE‐III, MMSE, MOCA, memory, attention, and language fluency, and that of left ATR was positively associated with ACE‐III, MMSE, MOCA, attention, and language scores (*p* < 0.05) (Figure 4B).

The FISO of left ATR and left IFO were negatively associated with ACE‐III, MMSE, MOCA, memory, attention, language fluency, language, and visuospatial function, and those of left SLF and FM were negatively associated with ACE‐III, MMSE, MOCA, memory, attention, language fluency, and language scores. The FISO of left ILF was negatively associated with ACE‐III, MMSE, MOCA, memory, attention, and language fluency, and that of left CgC was negatively associated with ACE‐III and memory score (*p* < 0.05) (Figure 4C).

Furthermore, no association was evidently found between other fiber bundle indexes and cognitive function (*p* > 0.05) (Figure 4A–C).

### 3.5 Association of aberrant NODDI indicators with cognition‐related plasma biomarkers (Sample cohort 2)

The ODI of left CgC was significantly positively associated with Aβ40, *p*‐tau181, GFAP, and NFL, and that of left TSLF was significantly positively associated with NFL. On the contrary, the ODI of left CgC was significantly negatively associated with Aβ42/Aβ40 and that of left ATR was significantly negatively associated with GFAP and NFL (*p* < 0.05) (Figure 4D).

The NDI of comprehensive abnormal fiber bundles was significantly associated with GFAP and NFL, and that of left IFO, left CgC, and FM was significantly associated with Aβ40 and Aβ42/Aβ40. Furthermore, the NDI of left CgC was significantly associated with *p*‐tau181 (*p* < 0.05) (Figure 4E).

The FISO of left IFO, left ATR, left ILF, left SLF, and FM was significantly associated with *p*‐tau181 and Aβ42/Aβ40, respectively. The FISO of left IFO, left ATR, ILF, left SLF, and FM was significantly associated with Aβ40, and that of left IFO, left ATR, ILF, left SLF, left CgC, and FM was with GFAP. Moreover, the FISO of left IFO, left ATR, ILF, left SLF, left UF, left CgC and FM was significantly associated with NFL (*p* < 0.05) (Figure 4F).

In addition, no association was observed between other fiber bundle indexes and cognition‐related plasma biomarkers (*p* > 0.05) (Figure 4D–F).

## 4 DISCUSSION

This NODDI study, together with TBSS and ROI‐based approaches, documented, for the first time, differences in WM integrity in patients with NOH, MOH, and SOH. We found marked abnormalities in the WM microstructure in patients with OH, especially in those with SOH. These abnormal WM microstructures were significantly associated with cognitive function. In addition, in the biomarker subsample, the abnormalities of these fiber bundles were associated with cognition‐related plasma biomarkers to varying degrees. Taken together, these findings suggest that the microstructural abnormalities of the WM may reflect the potential cognition‐related histopathological changes and that the decreased integrity of the above fiber bundles in MOH and SOH patients may be related to clinical cognitive impairment. These findings may provide novel insight into the diagnosis and treatment of OH‐related cognition.

In this study, we used a NODDI model that was very sensitive to WM microstructure to assess WM integrity. To depict the microstructure within the voxel, NODDI uses three parameters: NDI, ODI, and FISO, each with a scalar value ranging from 0 to 1. NDI and ODI are two key aspects of fractional anisotropy, the former being mainly used to estimate the volume fraction within the neurites and the latter to assess the degree of consistency in the direction of fiber distribution. FISO is used to quantify the volume of voxels occupied by free‐flowing cerebrospinal fluid.^19^ Despite a lack of consensus, cumulative studies have shown that the decrease in NDI and the increase in ODI and FISO may indicate a decrease in the microstructural integrity of the WM.^20^, ^21^, ^22^, ^23^

Most of the previous OH studies have focused on the volume of the gray matter structure and barely touched upon the microstructure of the WM.^24^, ^25^, ^26^ In this study, we found extensive microstructural anomalies in the WM in SOH patients, especially in FISO, which implies that the fiber bundles of the WM are likely predisposed to edema, necrosis, demyelination, and inflammation of nerve fibers. Interestingly, further analysis showed that the damage to the nerve fibers mainly occurred in the left WM and that an abnormality was present in the levels of NDI, ODI, and FISO in the left SLF, suggesting that the left SLF injury may indicate a typical change of the WM in OH patients.

The temporoparietal occipital region is a complex brain region through which a variety of WM fibers pass, mainly connecting fibers. The long connective fiber bundles of the WM are classified according to the cortical region of origin, including the SLF of the parietal lobe, the IFO of the occipital–parietal region, the UF of the temporal lobe, the ILF of the occipital‐temporal region, and the CgC.^27^, ^28^, ^29^ Although TBSS analysis showed no significant difference between NOH and MOH patients, the ROI analysis revealed that the ODI of the left CgC in the MOH group was reduced to varying degrees when compared with that of NOH and SOH counterparts, revealing a higher WM integrity of the left CgC in MOH than in NOH and SOH, which suggests that the left CgC may have a certain compensatory function in the pathological progression of OH and serve as an important target in the treatment of OH. This finding also highlights the importance of considering OH severity in investigating the abnormalities of the WM microstructure in OH patients. As an important part of the central autonomic neural network, CgC connects the frontal, parietal, and medial temporal lobes, as well as the subcortical nucleus and cingulate gyrus, thus playing a role in the regulation of cardiovascular function.^30^, ^31^ The role of CgC in blood pressure regulation has been documented many times and is well established.^32^, ^33^ In the current study, the findings strongly suggest an earlier degenerative pathology in the left CgC than in other WM structures during the course of OH, in which the destruction of WM fiber bundles may begin during the MOH stage and become more pronounced during the progression of MOH to SOH. Our study also found an association between NDI, ODI, and FISO values of the left CgC with the overall cognitive function and broad cognitive domains to varying degrees, especially in memory, which is consistent with the previous findings.^29^

In contrast, little literature is available to shed light on the decreased integrity of the UF, left ATR, SLF, and ILF, and its association with the cognitive function of OH patients. Our findings seem to open up a new research perspective for OH research. Changes in the integrity of the fibers (left ATR and ILF) in the regions associated with autonomic nervous function, such as the ponons, basal ganglia, and thalamus, further support the basic theory that OH is closely linked to the dysfunction of the autonomic regulatory regions. Autonomic cardiovascular function is regulated not only by the brain stem but also by different cortical regions. The SLF is thought to be the largest joint fiber tract system in the brain, which connects the marginal regions of the brain's hemispheres (frontal, temporal, and parietal lobes). The UF has traditionally been considered part of the limbic system and is usually divided into three parts: the temporal segment, the intermediate/insular segment, and the ventral/frontal extension.^34^ The temporal segment crosses from the entorhinal cortex, amygdala, and the anterior temporal lobe.^34^ The amygdala is involved in both sympathetic and parasympathetic regulation, and is closely related to the reflex and autonomic control of the heart and blood vessels.^35^ Therefore, we speculate that the damage to the UF and SLF in OH patients might result from the injured autonomic nervous control system. In the current study, the OH patients reported an alteration in the integrity of temporal WM fiber, which is associated with impaired baroreflex. The temporal insular cortex, which folds into the medial temporal lobe, is thought to be involved in the regulation of blood pressure and heart beats. The temporal insular cortex is also part of the reward system and the prominence network that controls behavior by inducing physical stimuli (such as thirst, heartbeat, visceral distention, temperature, and pain) and various cognitive processes. Previous studies have evidenced a close link between the temporal lobe and the left TSLF and the UF^34^ and temporal lobe atrophy in OH patients.^24^ Consistently, the current study also supported the regulatory role of the temporal lobe in OH patients.

Our study showed that the abnormal WM integrity in OH patients was mainly manifested in the left cerebral hemisphere. One possible explanation is that the right hemisphere has a greater blood supply than the left hemisphere,^36^ and is therefore less susceptible to damage by OH. However, due to the scarcity of studies on the abnormal WM asymmetry in MOH and SOH, the exact pathogenic mechanism remains to be resolved. Given that in the current study, FM was one of the most damaged areas in OH patients and that FM is part of the corpus callosum, the major joint WM fiber bundle that connects the left and right hemispheres,^37^ it follows that the SOH‐induced damage to WM may not be confined to just one hemisphere and that the adverse effects of OH may gradually spread from intra‐hemisphere to inter‐hemisphere, eventually leading to the widespread distribution of OH‐induced WM microstructural abnormalities in most cerebral regions. This notion has been confirmed by the findings of our study, showing that patients with SOH had extensive WM damage.

In addition, the damaged WM areas are also closely correlated with OH‐induced cognitive impairment. Although our study found no differences in cognitive function across the three groups, abnormal fiber bundles in the WM of the MOH and SOH groups suggest underlying impairment in the cognitive microstructure and brain function of OH patients. Our study showed that the WM tract, severely damaged in OH patients, was significantly associated with clinical cognitive functions. A host of studies have documented the association of ATR, IFOF, ILF, and SLF with memory,^38^ that of FM and ATR with executive function,^39^ the role of ILF in visual processing and language understanding,^40^, ^41^ the putative role of UF in episodic memory, language and social emotional processing,^34^ and that of CgC in episodic memory and executive function.^29^ In a similar line, the damaged WM area is also associated with OH‐induced neurological diseases^29^, ^42^, ^43^, ^44^ and with cognition‐related plasma biomarkers,^45^, ^46^, ^47^ which also supports that OH contributes to the occurrence of cognitive diseases.^48^, ^49^, ^50^

The association between OH and WM microstructure abnormalities can be explained by several potential mechanisms. OH is prone to recurrent cerebral hypoperfusion,^51^, ^52^, ^53^ resulting in chronic cerebral ischemia and exacerbated oxidative stress, which ultimately induces endothelial dysfunction and destruction of the blood–brain barrier.^54^ The former promotes pro‐inflammatory and pro‐coagulant states, while the latter leads to the thickening and disintegration of small blood vessel walls. In addition, OH can cause hardening of the arteries.^55^ All these pathogenic factors and pathways can further compromise the blood supply to the brain. A previous study has reported that the brain is highly sensitive to insufficient blood supply, which can lead to the breakdown of the microstructural integrity of the WM,^56^, ^57^ including ischemia, demyelination, axon damage, inflammation, or edema. Alternatively, the damage to the WM microstructure may also precede the OH onset, that is, the damage of WM may impair the blood pressure regulation and the autonomic neural network, ultimately triggering the onset of OH.

Strengths of our study included the recruitment of subjects with complete clinical, neuropsychological data, and plasma biomarker data, which were integrated with DWI data. Hence, we were able to investigate the differences in the microstructures of the WM in NOH, MOH, and SOH patients, and the association of these differences with cognition and cognition‐related biomarkers. In addition, our study applied advanced diffusion imaging techniques with 3 T MR tomography for NODDI, which provides finer microstructural indicators of tissue than diffusion‐weighted imaging^20^, ^58^ and facilitates a detailed and accurate assessment of the WM integrity.

However, certain limitations remain in our study. Owing to its cross‐sectional design, selective survival bias was inevitable. In addition, the association between WM integrity and cognition‐related plasma biomarkers was investigated in a small sample, although the population was randomly selected to reduce statistical error. Finally, the results are derived from a Chinese elderly population and caution needs to be taken when they are generalized to other populations.

In conclusion, our study evidences that abnormalities in the WM microstructure may manifest early in MOH patients, which are closely associated with a wide range of cognitive domains and cognition‐related plasma biomarkers, and that CgC may be a key target for early intervention of OH. These findings suggest that the abnormalities of the WM microstructure may serve as early indicators of OH‐related cognitive dysfunction and early intervention should initiated to curb the progression of MOH to SOH.

## AUTHOR CONTRIBUTIONS

Yingzhe Cheng and Yanping Wang performed the study, participated in the protocol design, collected and analyzed the data, and wrote the main text; Xiaodong Pan was responsible for funding acquisition and process supervision, and participated in various relevant discussions as the project leader; Lin Lin and Shaofan Jiang were responsible for MR data visualization; Peilin Huang and Jiejun Zhang helped recruit patients. All authors participated in writing the manuscript and approved the final version of the manuscript.

## FUNDING INFORMATION

This work was supported by the Joint Funds for Innovation of Science and Technology, Fujian Province [grant number: 2021Y9037]; the National Clinical Key Special Subject of China; Financial Special Project of Fujian Province [grant number: 2021XH009]; and the Guiding Project of Fujian Province [grant number: 2021Y0020].

## CONFLICT OF INTEREST STATEMENT

The authors declare no conflicts of interest related to the work presented here.

## PATIENT CONSENT STATEMENT

All participants provided written informed consent at the time of study registration and DWI data collection. Informed consent for participants with severe cognitive impairment was obtained from their guardians.

## Data Availability

The data that support the findings of this study are available from the corresponding author upon reasonable request.
